# Promoting Physical Activity and Preventing Falls Among Older Adults in a Nursing Home Setting: Protocol for Development and Implementation of the BeSt Age Mobile App

**DOI:** 10.2196/74174

**Published:** 2025-10-06

**Authors:** Janina Krell-Roesch, Jonathan Diener, Jelena Krafft, Sabine Rayling, Alexander Woll, Kathrin Wunsch

**Affiliations:** 1 Institute of Sports and Sports Science, Karlsruhe Institute of Technology, Kaiserstr. 12, Karlsruhe, 76131, Germany, 49 72160841664

**Keywords:** mobile health, physical activity, fall prevention, older adults, nursing homes, cluster randomized controlled trial

## Abstract

**Background:**

Most nursing home residents do not meet physical activity guidelines. Many interventions to promote physical activity and prevent falls in nursing home residents have low adherence rates, lack a theoretical foundation, or require much time from staff for preparation or delivery.

**Objective:**

This study aims to describe the rationale, development, and implementation approach of the BeSt Age app through a cluster randomized controlled trial. We also provide baseline characteristics of the study sample and discuss possible implications for further app developments.

**Methods:**

We iteratively developed a novel, tablet-based mobile app (BeSt Age) that enables nursing home staff to deliver individualized physical exercise training to residents with or without motor or cognitive impairments. The app was designed and developed based on an intervention-mapping approach. A needs assessment was performed, followed by defining objectives, theory-based methods, program development, implementation, and evaluation. We took several steps to ensure that the app was based on a sound theoretical background and considered limitations identified in prior research. For implementation and evaluation purposes, we conducted a study among 229 older adults from 19 nursing homes (171 females, 58 males; mean age 85, SD 7 years). Results will be used to examine the effectiveness of the app with regard to different outcomes. Primary outcomes among participating nursing home residents are quality of life, fall risk evaluated through 2 performance-oriented balance tests, and fall incidence. Secondary outcomes include motor performance, cognition, activities of daily living, physical activity behavior, and fall efficacy. In this paper, we examined differences between intervention group (IG) and control group (CG) participants at baseline using the chi-square test, the Mann-Whitney *U* test, or the *t* test.

**Results:**

The IG (n=137 from 11 nursing homes) received a 12-week intervention with the BeSt Age app in small, homogenous groups of 5‐7 nursing home residents, with 2 exercise sessions per week, each lasting 25‐30 minutes. The CG (n=92 from 8 nursing homes) received usual care. At baseline, the IG had a statistically significantly larger number of females, participants had a higher BMI, and more participants rated attending physical activity programs as important. There were no further statistically significant differences between the groups. Results with regard to the effectiveness of the BeSt Age app are expected to be published in spring 2026.

**Conclusions:**

If proven effective, the BeSt Age app may be a viable solution for physical activity promotion and fall prevention among older adults residing in nursing homes, thereby contributing to maintaining quality of life and overall well-being in this vulnerable population. The app can support nursing home staff in delivering exercise training to residents with minimal additional workload and without requiring specific resources.

## Introduction

Regular engagement in physical activity from young adulthood through middle into old adulthood is of high importance for an independent, self-determined life and maintained functional performance [1]. Research has shown that physical activity has various beneficial effects on physical as well as mental health and well-being in older adults [2] and may delay age-related cognitive decline [3]. Physical activity is also associated with a decreased risk of falls in community-dwelling older adults [4] and nursing home residents [5,6], albeit conflicting results for the latter group have been reported [7]. Nevertheless, it is well established that falls are very common among individuals residing in nursing homes [8] and are associated with adverse health outcomes, such as increased risk of morbidity and mortality [9].

The World Health Organization recommends 150-300 minutes of moderate- to high-intensity aerobic physical activity per week for adults and moderate strength training on 2 or more days per week that involves all major muscle groups of upper and lower extremities [10]. In addition, with regard to fall prevention, persons aged 65 years and older should perform activities that can improve functional balance and proprioception on at least 3 days per week [10]. A minimum of 4600 steps per day, averaged over a week of free-living behavior, has been recommended for older adults by an expert group [11]. Furthermore, another expert task force from the International Association of Gerontology and Geriatrics—Global Aging Research Network and the European Region Clinical Section has published physical activity recommendations for older adults residing in long-term care facilities [12]. They defined 2 different levels of recommendations, that is, a first set aiming at reducing sedentary behaviors for all residents in long-term care facilities and a second set aiming to establish specific, evidence-based guidelines for exercise training for well-defined subgroups of residents. To this end, they recommend multicomponent training, particularly focusing on strength, aerobic endurance, balance, and flexibility. This training should be of moderate intensity, carried out at a frequency of at least twice a week, and with a duration of 35-45 minutes per session.

However, despite the widely acknowledged benefits of physical activity, and despite many individuals having intentions to engage in physical activity, most fail to follow through, a phenomenon referred to by sports psychologists as the intention-behavior gap [13]. It is well known that older adults do not engage in a sufficient amount of physical activity, and this is particularly true for those residing in care facilities. For example, a literature review showed that care facility residents only engage in low levels and intensities of physical activity, with numbers of steps per day averaging between 385 and 3387 across studies, and that there is very little variation in movement patterns [14]. Another review reported daily sedentary times of 11.6 hours among residents in high-level care facilities, such as nursing homes [15]. Furthermore, a study conducted in German nursing homes using sensor-based assessment revealed that residents’ movement space was mainly restricted to their personal rooms and the adjoining residential units during 90% of the daytime [16].

This lack of physical activity among nursing home residents is alarming, particularly since the negative consequences of sedentariness may be accelerated by age-related physical and cognitive decline, thereby further increasing care dependency and burden for caregivers and family members [17]. Different factors have been discussed in the literature that may impact physical inactivity and sedentariness. In community-dwelling older adults [18], main barriers to physical activity include personal characteristics of older adults (eg, poor physical and mental health status and lack of interest) and interpersonal aspects, such as lack of company or social network. For older adults residing in nursing and care homes [19,20], additional barriers may include physical environment (eg, lack of access to green spaces) or organizational barriers, such as nursing home staff shortages, time constraints, or insufficient communication of available exercise, as well as low awareness among residents. Recently, a systematic literature review of caregiver-provided exercise interventions for individuals with dementia residing in nursing homes reported that the main barriers to the implementation of interventions, such as physical exercise, care-integrated physical activity, outdoor or walking activity, technology-supported physical exercise, and dancing were a lack of knowledge about positive effects of physical activity, refusal of residents with dementia to participate, or insufficient time and resources for implementation among caregivers [21], albeit also acknowledging that all stakeholders considered physical activity interventions as useful and relevant.

To date, a growing body of interventions and programs aimed at promoting physical activity, preventing falls, or improving motor performance is being more widely implemented in care settings, and different literature reviews are available focusing on the effectiveness of such programs on various outcomes of interest, for example, quality of life and related factors in older adults in residential care facilities [22] or mental health, such as well-being and cognition in older persons residing in nursing homes [23]. However, there are still several challenges that need to be addressed. One challenge, for example, is low adherence. To this end, a French cross-sectional study revealed that only 1914 of 5402 older nursing home residents from a total of 163 different nursing homes participated in physical activity programs provided by their nursing homes and that classes were only offered once per week or less with a median duration of 45 (IQR 30-60) minutes, on average [24]. Of note, participation as well as exercise frequency and levels were increased among nursing home residents when an exercise instructor was present [24]. Another limitation is a lack of theory-based or derived interventions, such as interventions incorporating behavior change techniques. While a meta-analysis including 171 studies revealed no differences in overall effect sizes between theory-based and no-stated-theory interventions, it provided evidence that theory-based interventions incorporated a greater number of behavior change technique clusters, which may in turn be associated with better effects [25]. With regard to older adults in particular, a review examined behavior change techniques in interventions aimed at increasing physical activity using the theory of planned behavior. The investigators identified only 7 studies, and the most frequently used techniques were goal setting, action planning, and credible sources [26]. Another review including 70 physical activity interventions identified action planning, instructions on how to perform a behavior, graded tasks, and demonstration of behavior as the most common behavior change techniques and also showed that interventions carried out in older persons’ homes used 9 behavior change techniques, whereas interventions conducted in a residential care facility used only 6 behavior change techniques on average [27]. Recently, researchers from Taiwan developed a motivation theory-based walking intervention and reported that it enhanced the self-efficacy of 30 older adults living in long-term care facilities and was perceived as helpful in increasing physical activity engagement [28]. Further limitations, as also mentioned above, include but are not limited to lack of knowledge among caregivers along with insufficient time and resources for implementation of physical exercise in nursing homes [20,21] or the use of “one-fits-all” physical exercise programs. To this end, it has been postulated that individualized exercise programs that are deliberately tailored to the needs and preferences of older adults residing in nursing homes may have higher effectiveness on physiological outcomes [29], variables related to brain health [30], physical activity [31], physical performance [32], or gait [33].

In light of these limitations and challenges of physical exercise implementation in care settings, different approaches and solutions have been discussed in the literature. Those include nontechnology-based approaches, such as exercise delivered by trained nursing home staff [34,35] or focus on environmental aspects that may impact physical activity in nursing home residents [36], as well as technology-based approaches, such as responsive surface technology [37], virtual reality–based systems [38,39], exergaming and other mobile health and eHealth–based interventions [40], and even social robots [41]. Indeed, one promising approach that is also rather simple to both develop and implement, cost-effective, and close to real life may be the use of mobile apps. Research has shown that digital, touchscreen-based technologies may be effectively used for person-centered care and engagement of nursing home residents [42], and can also support caregivers who may not have prior expertise or knowledge on physical activity training, or only have very limited time during their work routines in delivering exercise interventions to nursing home residents [43]. Moreover, a study examined the usability as well as the effects of an app-derived physical activity intervention for older adults with dementia residing in nursing homes, but the validity of results was limited by the small study sample size [43,44]. Other studies have shown that nursing staff are willing to use digital and assistive technologies, including mobile apps, but that access to such technologies, including Wi-Fi, is limited in nursing home settings, and that in order to maximize acceptance, it is crucial to involve nursing staff in the development process and provide adequate training and testing opportunities tailored to their experience and affinity for technology [43,45,46]. Overall, even though preliminary results regarding usability, acceptability, or effectiveness are promising, digital interventions for physical activity promotion in older adults remain understudied, and existing solutions are too heterogeneous for meaningful comparison of results [47], thereby limiting evidence. Furthermore, little is known about the practical application [48], accessibility, acceptability, and sustainability of such solutions [49], and many care institutions are thus still hesitant to adopt these innovations [50,51].

To address some of the challenges and limitations related to physical exercise implementation in care settings as outlined above, we have iteratively developed and implemented a mobile app called the BeSt Age app and examined its effectiveness in a cluster randomized controlled trial (cRCT). The aim of this paper is to describe the rationale, development steps, and implementation of the BeSt Age app, as well as to provide an overview of baseline characteristics of the cRCT study sample and discuss future implications.

## Methods

### Rationale for and Development of the BeSt Age App

To address gaps in prior research outlined above, the tablet-based BeSt Age app was designed with the overarching aim of supporting care staff in delivering an individualized physical exercise program to small, homogeneous groups of nursing home residents. Based on an intervention-mapping approach [52], a needs assessment was performed, followed by defining objectives, theory-based methods, program development, implementation, and evaluation. Thus, we took the following steps to ensure that the app was designed and developed based on a sound theoretical background and considered results and limitations identified in prior empirical research.

First, we conducted an online survey with 200 nursing home staff across Germany to examine previous experiences and willingness to use digital solutions, including but not limited to the purpose of physical activity promotion and fall prevention, as well as perceived possible risks, attitudes, and expectations in this regard [45,46]. We also inquired about affinity for technology or technical equipment available in nursing homes. We then conducted an interview study with stakeholders (ie, three managers, six employees providing care for residents, and ten residents) from seven different nursing homes to gather information about existing physical activity programs that they may have implemented in their institutions and to identify prior experience, needs, expectations, fears, and possible barriers to using digital technology. The results from both the internet-based survey and interview study were used to ensure that our app would have a high acceptability and feasibility.

Second, we defined the objectives of the BeSt Age app. Those included (1) the delivery of physical exercise tailored to the needs and preferences of nursing home residents (rather than “one-fits-all” exercise) by nursing home staff; (2) creation of small groups of residents with comparable motor and cognitive performance status using an in-built algorithm; (3) inclusion of a wide range of nursing home residents, for example, also those using a wheelchair or walking aids (we only excluded persons with very severe motor, neurological, or cognitive impairments and disorders); and (4) a multimodal exercise program, including both motor and cognitive stimulation.

Third, we took several theoretical considerations into account that have been the foundation of lifestyle interventions aimed at improving health-related outcomes, such as social cognitive theory [53], ensuring that our app is evidence-based, comprehensive, and tailored to meet the objectives as outlined above. Furthermore, app content was developed based on the self-determination theory [54], as it fosters intrinsic motivation by addressing three fundamental psychological needs, that is, autonomy (users choose activities), competence (activities are tailored to ability levels), and relatedness (social support through group activities). Also, the COM-B (Capability, Opportunity, Motivation, and Behavior) model [55] was considered for app development. According to this model, behavior change depends on capability (physical and psychological), opportunity (social and physical environment), and motivation. We designed the app in a way that it would target all 3 components by simplifying exercise selection, embedding social elements, and using gamification features to enhance motivation. Moreover, basic theories on enhancing acceptance of technology, such as the Technology Acceptance Model [56], as well as published physical activity recommendations for older adults residing in long-term care facilities [12], were taken into account.

Fourth, regarding the exercise program development, we drew on our expertise from the InCoPE app (Individualized Cognitive and Physical Exercise; Institute of Sports and Sports Science, Karlsruhe Institute of Technology) that our group had developed and evaluated prior to the BeSt Age app. Briefly, the InCoPE app was designed to enable nursing home staff to deliver an individualized exercise program to small, homogenous groups of nursing home residents with dementia [43,44,57]. In contrast, the BeSt Age app was developed to enable nursing home staff to deliver an individualized exercise program to nursing home residents regardless of their cognitive impairment status. Thus, we consider the BeSt Age to be more inclusive than our previously developed InCoPE app. When designing and developing the BeSt Age app, we also considered results from a systematic literature review performed by our group, which examined the effectiveness and acceptability of eHealth and mobile health interventions to promote physical activity and prevent falls in nursing homes [40].

As a fifth and last step, evaluation planning involved the design of a cRCT to examine the effectiveness of the app with regard to different outcomes of interest. Subsequently, the app prototype was developed and tested in a 2-week pilot study in 2 nursing homes to examine its feasibility, usability, and user experience. Feedback obtained during the pilot study was incorporated into the final version of the app, which was then used in the cRCT.

To conclude, we developed the app in an iterative manner, that is, the app was continuously revised and improved based on the several steps we took, to ensure that the final version would fulfill both expectations and needs of the users, that is, nursing home staff and residents. We adopted a 3-component user-centered design strategy to guide the development of our interface, and this approach yielded comprehensive user feedback through direct stakeholder engagement and contextual testing in the target environment. First, our interview study provided us with insights on workflow integration, usability requirements, and practical implementation considerations, which informed our initial design decisions and feature prioritization. Second, we collaborated with a usability and user experience expert from the software company that developed the app to ensure adherence to established usability principles and best practices for apps serving older adults. Third, the pilot study allowed us to observe actual user interactions, identify usability issues, and collect feedback from both staff and residents in authentic care settings, and it also enabled us to validate and refine the interface based on actual usage patterns.

### Features and Functionality of the BeSt Age App

In general, as mentioned above, the purpose of the app is to assist nursing home staff in delivering individualized exercise training programs to small, homogenous groups of nursing home residents. Training with a single individual is also possible but less feasible given the restricted time and personnel resources in many care facilities [44]. A distinctive feature of the app is that it takes the cognitive and motor performance status of an individual into account, that is, exercises that align with the motor and cognitive capabilities of the small group of residents are selected through a distinctively developed algorithm. To this end, the app contains a large selection of about 150 different exercises and movements, and also different variations for most of them in terms of intensity or motor requirements needed to perform the exercise, thereby allowing for maximal variety in the training. Furthermore, the app creates a detailed plan for each exercise session, thereby reducing the workload of staff for preparing and providing exercise sessions.

The app workflow requires nursing home staff to create an overall profile for their facility (eg, by providing the name and indicating which materials are available) as well as a personal profile for each resident by inserting subjective information, as outlined in Textbox 1.

Textbox 1. **Subjective information.**

**Motor performance (multiple selection)**

- The person is able to get up from a chair with or without aids.
- The person is able to walk with or without aids.
- The person is not able to get up from a chair or walk.

**Cognitive performance level (single selection)**

- The person has no or little cognitive impairment.
- The person has moderate cognitive impairment.
- The person has severe cognitive impairment.

**Personal exercise preferences (multiple selection)**

- The person likes to dance or exercise with music.
- The person likes to sing.
- The person likes to exercise with balls.

Once the profiles for all residents are created, matching algorithms implemented in the app create small, homogeneous groups of residents (up to 7 persons), with similar motor and cognitive performance status as well as personal exercise preferences. To this end, residents’ characteristics and interests are matched by the algorithm with the requirements defined for each exercise (eg, the person must be able to stand for this exercise) and the materials available in the respective care facility.

Briefly, the matching algorithm was developed based on systematic feedback from nursing home staff collected during our interview study and pilot testing. It incorporates practical considerations identified by nursing home staff regarding the delivery of physical activity sessions. To validate its effectiveness, we tested the algorithm using simulated resident profiles with varying functional levels and confirmed that it successfully creates homogeneous groups. The app also features a calendar function, which can be used by nursing home staff to plan and schedule exercise sessions with different groups of residents for the entire week, month, or period of interest.

On the day of a given exercise session, nursing home staff operate the tablet and will be guided step-by-step through the entire exercise training program by following the on-screen instructions. On the starting screen, they are first shown an overview of all exercises included in the entire session, as well as any materials (eg, balls, chairs, and balloons) that may be needed, so that they can prepare the materials in advance. Once the program is started, each exercise will be shown and described in detail on a new screen, containing information about the name and purpose of the current exercise, any material needed (eg, a chair), potential risks or side effects (eg, pain), and duration or number of repetitions (eg, 2 times of 20 repetitions). A brief video clip showing an older person carrying out the exercise is also provided on the screen. In addition, the exercise program is accompanied by background music that can be turned on and off as needed.

**Figure 1.**
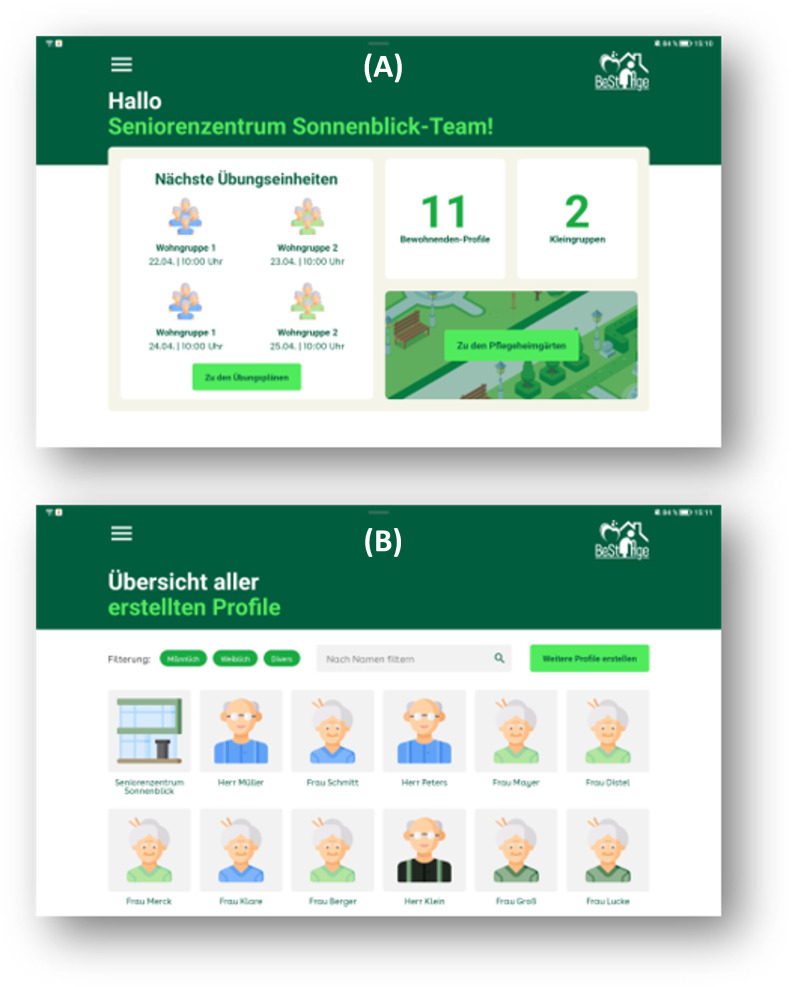
Profiles of facilities and individuals. (A) Overall facility profile showing an overview of participants, training groups, and date and time of next training sessions. (B) Overview of individual profiles of participants with integrated search and filter function.

**Figure 2.**
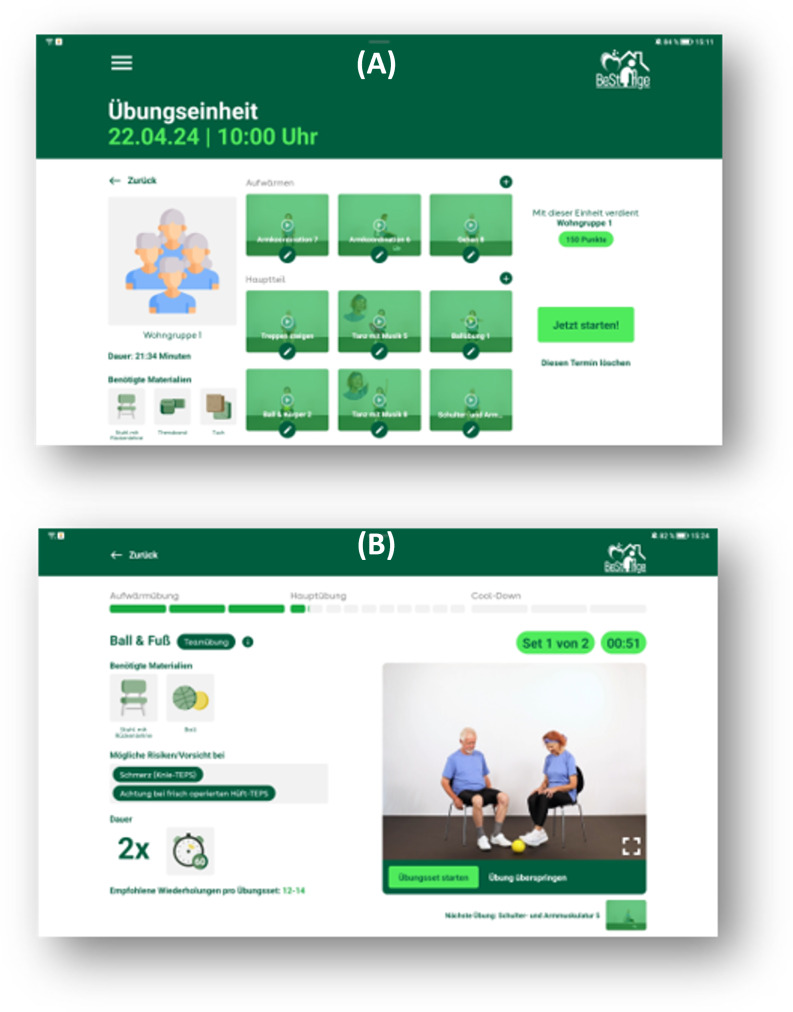
Training sessions. (A) Overview of a training session including participants, duration of the session, material needed, and planned exercises. (B) Exercise screen, which includes material needed, possible risks, duration of the exercise, suggested repetitions, and a video of the exercise execution.

**Figure 3.**
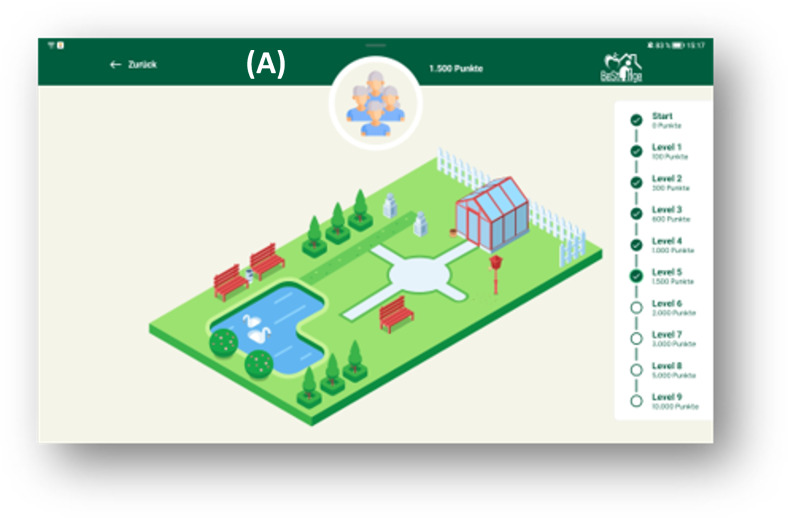
Gamification within the BeSt Age app. (A) Nursing home garden as gamification element.

The main exercise program contains 2-3 exercises for warm-up (ie, mobilization and coordinative exercises), 5-7 main exercises (ie, strength, balance, endurance, and gross motor coordination), and 2 exercises for cooldown (ie, relaxation and body perception), and takes approximately 25-30 minutes to complete. Please refer to Multimedia Appendix 1 for examples of warm-up, main, and cooldown exercises. The algorithm is trained to provide well-balanced sessions, including a comparable number of exercises for the strength of different body parts, mobility, and balance. If needed, nursing home staff can adapt the exercise sessions by skipping exercises while using the app (also, exercises can be removed or added before the program has been started on the app). After each session, nursing home staff can rate each exercise using a feedback feature. This feedback is also used to ensure progression with regard to intensity by suggesting more challenging versions of the exercises in upcoming sessions if and when an exercise has been rated positively several times. If an exercise is rated poorly, the app will exclude it and similar exercises from further sessions for this group. Furthermore, attendance of all individuals by group can be documented after the session, with the option of choosing reasons for not attending the program. Nursing home staff can also review the description of the upcoming session to identify any required materials and prepare them in advance. Of note, the app also features a gamification element for motivational purposes, that is, the group is awarded credit points for completed sessions, which can be used to purchase new items (eg, a bench and flowers) for a digital nursing home garden. Please refer to the screenshots (Figures 1-3) showcasing the main features of the app.

Overall, the following behavior change techniques were integrated into the app: (1) information on health, social, and environmental consequences, that is, health-related educational content, was presented to participants prior to each exercise session to enhance knowledge of fall prevention benefits; (2) demonstration of behavior, that is, video-based exercise demonstrations; (3) social comparison with other groups, that is, progress visualization via the digital nursing home garden enabled participants to compare their group’s achievement with other participating groups; (4) prompts and cues, that is, audio-based reminder notifications were programmed to occur 15 minutes prior to scheduled exercise sessions; (5) habit formation, that is, the built-in calendar scheduling function enabled nursing home staff to establish consistent exercise sessions twice weekly at standardized times and days; (6) nonspecific reward, that is, a point-based reward system provided immediate positive reinforcement following completion of exercise sessions. Also, the app was designed to accommodate residents with varying degrees of cognitive impairment through several adaptive features. For example, educational content and exercise demonstrations used clear, simplified language and visual cues to enhance comprehension for persons with cognitive limitations. Given the cognitive challenges of the target population, nursing home staff served as primary facilitators and operated the app interface while guiding residents through the exercises, thereby reducing cognitive load on participants. In addition, audio prompts were combined with visual notifications to accommodate different cognitive processing abilities and ensure message reception across varying impairment levels. Furthermore, the digital garden visualization provided concrete, easily interpretable feedback that residents could understand regardless of cognitive status, with staff providing additional verbal explanations as needed. Also, the consistent scheduling and habit formation features embedded in the app were particularly beneficial for cognitively impaired residents, as establishing routines may compensate for memory deficits and reduce cognitive demands.

### Implementation Approach: Cluster Randomized Controlled Trial

To test the effectiveness and acceptability of the BeSt Age app, we carried out a 12-week cRCT. Randomization into an intervention group and control group was done at the facility level in order to ensure a sufficient number of participants that would allow for the in-built matching algorithm to form homogenous groups for the exercise training program. To this end, a list of nursing homes in the city of Karlsruhe and the surrounding districts in the German state of Baden-Wuerttemberg was created, and we contacted the nursing homes by telephone and provided on-site informational meetings. Allocation of nursing homes to either the intervention or control group was performed using a computer-generated randomization procedure. If possible, investigators assessing gait, motor, and cognitive performance outcomes were blinded to group allocation. Blinding of participants or nursing home staff delivering the intervention was not possible.

### Study Sample

An a priori power analysis was conducted using G*Power (version 3.1.9.7; Heinrich-Heine-Universität Düsseldorf [58]), based on the assumption that we would calculate 2-factor ANOVAs with repeated measurements, 2 groups, 2 measurements, *α*=.05, 1-β=.80, and ɳ²=0.02, and revealed a required total sample size of 100 participants. The effect size (ɳ²=0.02) used for the calculation of the required sample size was based on results presented in our literature review [40]. However, we will decide on the appropriate statistical approach based on the intraclass correlation coefficient (ICC) derived from the original data once data collection is completed. In the literature [59], an ICC of ≥0.05 has been suggested as a cutoff value for determining whether to use multilevel modeling. Since statistically significant differences between groups (eg, with regard to sex distribution, BMI, or perceived importance of physical activity) may confound outcomes, we will also consider those variables as covariates and adjust future analyses accordingly. Furthermore, in case of data missing at random according to the Little test, we plan to include multiple imputation procedures. To this end, we will use SPSS’s (IBM Corp) multiple imputation procedure to generate multiple imputed datasets (typically 5‐10 imputations) using the fully conditional specification method, which is appropriate for our dataset. Considering an estimated dropout rate of 20%, anticipated low adherence to the intervention, and adjustment to cluster randomization using a variance inflation factor [60], the sample size was set to 255 participants. The sample size calculation based on a priori power analysis was designed to detect overall intervention effects at the facility level rather than within specific subgroups, for example, based on cognitive function or mobility levels; however, subgroup analyses may be performed when suitable.

We defined the following inclusion criteria for nursing home residents: aged >65 years; ability to follow verbal instructions; and at least 50% functional capacity of extremities; that is, participants were required to have functional use of at least 50% of their extremities (at least 2 of 4 limbs: both arms, both legs, or 1 arm and 1 leg). We chose this criterion to ensure that the intervention would be rather inclusive, allowing, for example, wheelchair users to participate. Exclusion criteria were very severe cognitive, neurological, or motor impairments or disorders. We deliberately did not formulate any additional exclusion criteria in an attempt to be rather inclusive, particularly since prior studies had defined more strict inclusion and exclusion criteria that may not apply to many residents of nursing homes and may not be reflective of a real-world scenario [40]. Eligibility of participants was verified at baseline assessment and based on the aforementioned predefined criteria.

### Intervention and Outcome Measures

**Figure 4.**
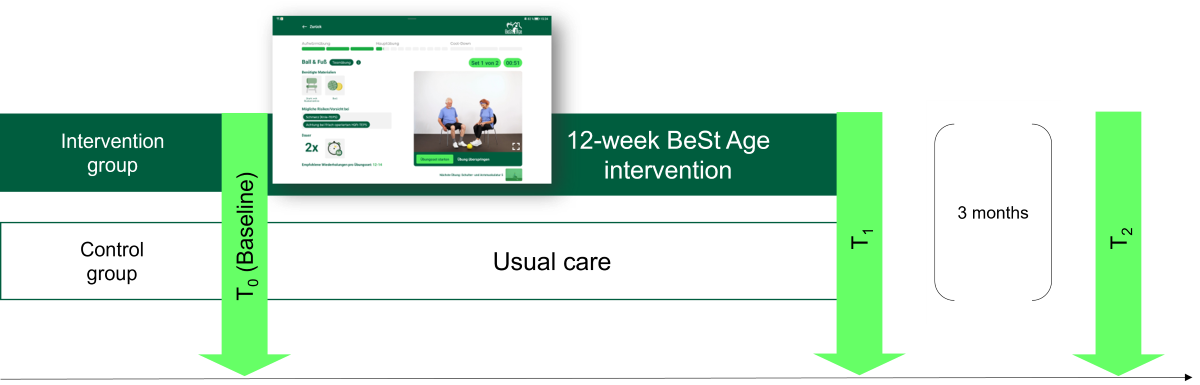
Study design.

The intervention group received a 12-week intervention with the BeSt Age app (including features as described above) in small, homogenous groups of about 5-7 participants, on average, with 2 exercise sessions per week, each lasting about 25‐30 minutes. The control group received usual care. Baseline (T0) and postintervention (T1) assessments were carried out, and a follow-up survey (T2) was conducted 3 months after the end of the intervention to assess fall incidence. To ensure protocol adherence, all nursing home staff involved in the intervention received standardized training sessions covering app functionality, exercise session protocols, resident engagement strategies, and data collection procedures before the cRCT. Training materials also included detailed protocol manuals to ensure uniform understanding across facilities. To monitor intervention fidelity, we maintained regular contact with facility coordinators and provided ongoing technical support to address implementation issues. App usage analytics also provided objective data on session completion rates, feature usage, and timing of interventions across facilities. Furthermore, all assessments as part of the study were conducted and administered to nursing home residents and staff by trained members of the research team, and we also provided them with a detailed manual to enhance standardization of assessment and scoring procedures. Also, if and when possible, we assigned the same persons to administer the same tests in different nursing homes to ensure consistency in administration and scoring. Finally, our data handling procedures ensured full compliance with the European Union General Data Protection Regulation, including appropriate consent procedures and secure local storage protocols. All participant data were stored exclusively on local tablets without cloud-based storage or remote server connections, thereby eliminating risks associated with data transmission. Tablets were operated without internet connectivity, ensuring complete network isolation and preventing unauthorized external access to participant data. Please refer to Figure 4 for a graphical display of the study design. Please refer to Table 1 for an overview of all primary and secondary outcomes assessed at T_0_, T_1_, and T_2._

**Table 1.** Overview of primary and secondary outcomes.

Dimension	Assessment	Time of measurement			Calculated variable or score used for analysis
		T_0_	T_1_	T_2_	
Primary outcomes					
Quality of life	EUROHIS-QOL^a^ questionnaire [61,62]	NHR^b^	NHR	N/A^c^	Total score (8-40)
Fall risk (performance-oriented balance tests)	Timed Up and Go [63]	NHR	NHR	N/A	Time (seconds)
	Berg Balance Scale [64]	NHR	NHR	N/A	Total score (0-56)
Falls	Fall incidence [65]	NHR	NHR	NHR	Number of falls
Secondary outcomes					
Grip strength	Digital hand dynamometer [66]	NHR	NHR	N/A	Best out of 6 trials in kg
Arm strength	30-seconds arm curl test [67]	NHR	NHR	N/A	Number of arm curls in 30 seconds
Walking speed	10-meter walking test [68]	NHR	NHR	N/A	m/s
Cognitive status	Montreal Cognitive Assessment [69,70]	NHR	NHR	N/A	Total score (0-30)
Anticipatory motor planning	Bar-transport task [71]	NHR	NHR	N/A	Number and percentage of end-state comfort grasps
Activities of daily living	Katz Index [72]	NHR	NHR	N/A	Total score (0-6)
Physical activity behavior	Life-Space Assessment in Institutionalized Settings (LSA-IS) [73,74]	NHR	NHR	N/A	Total score (0-90)
Fear of falling	7-item Short Falls Efficacy Scale-International [75]	NHR	NHR	N/A	Total score (7-28)
Digital competence	Single-item (Likert-Scale format)	NHR	NHR	N/A	Total score (1-5)
Reasons for and motivation to participate in physical activity in general	Open-ended questions	NHR	N/A	N/A	N/A
Motivation to continue using BeSt Age app	Open-ended questions	N/A	NHR	N/A	N/A
Influence of gamification	Open-ended questions	N/A	NHR	N/A	N/A
Reasons and motivation to work with BeSt Age app	Open-ended questions	NHS^d^	N/A	N/A	N/A
Usability of BeSt Age app	System Usability Scale [76]	N/A	NHS	N/A	Total score (0-100)
User experience	AttrakDiff [77]	N/A	NHS	N/A	4 dimensions scored –3 to 3
App usage	User statistics derived from app data	N/A	NHS	N/A	N/A
Enjoyment	Open-ended questions	N/A	NHS	N/A	N/A
Future app use	Open-ended question	N/A	NHS	N/A	N/A

a EUROHIS-QOL: European Health Interview Survey Quality of Life.

b NHR: nursing home residents.

c Not applicable.

d NHS: nursing home staff.

Primary outcomes of interest among participating nursing home residents included quality of life assessed using the 8-item European Health Interview Survey Quality of Life (EUROHIS-QOL) questionnaire (calculated variable: total score ranging from 8 to 40 with a lower score indicating worse quality of life) [61,62] and fall risk evaluated through 2 performance-oriented balance tests, that is, the Timed Up and Go Test assessing lower extremity function and mobility (calculated variable: time in seconds to get up from chair, walk 3 meters, turn around, return 3 meters, and sit back down at chair; walking aids such as canes or rollators were permitted) [63] and the Berg Balance Scale assessing static and dynamic balance through a 14-item test battery, with each item rated from 0 to 4. Here, a lower score indicated greater balance impairment (calculated variable: total score ranging from 0 to 56) [64]. We also recorded fall incidence from nursing home staff at 6 months before the intervention, during the 12-week intervention period, and during the 3-month follow-up period postintervention. To this end, a fall was defined as “an event in which the individual unintentionally comes to rest on the ground or another lower level” according to the “Expert Standard for Fall Prevention in Nursing Care” of the German Network for Quality Development in Nursing Care [65].

Secondary outcomes of the cRCT included 3 motor performance tests, including grip strength assessed using a digital hand dynamometer according to the Southampton Protocol (calculated variable: best out of 6 trials, that is, 3 for each hand, in kg) [66], a 30-second arm curl test (calculated variable: number of performed arm curls in 30 seconds) [67], and a 10-meter walking test (2 trials at preferred pace; 2 trials at fast pace; walking aids such as canes or rollators were permitted) [68]. Additional outcomes included cognitive status assessed using the Montreal Cognitive Assessment (MoCA; calculated variable: score ranging from 0 to 30, with lower scores indicating greater cognitive impairment) [69,70], the end-state comfort effect as an indicator of anticipatory motor planning using the bar-transport task (calculated variables: number and percentage of end-state comfort trials in uncritical and critical conditions) [71], activities of daily living assessed using the Katz Index (calculated variable: score ranging from 0 to 6, with lower scores indicating lower function and dependence) [72], life space or physical activity behavior assessed using the Life-Space Assessment in Institutionalized Settings (LSA-IS; [73]) questionnaire (calculated variable: score ranging from 0 to 90 with a lower score indicating a greater degree of immobility) [74], and fear of falling which was assessed with the 7-item Short Falls Efficacy Scale-International (calculated variable: score ranging from 7 to 28, with higher scores indicating more severe concern about falling) [75]. All participants also rated their digital competence, and we created a score ranging from 1 (very low) to 5 (very high). In addition, participants were asked various questions, for example, about their reasons and motivation to participate in physical activity programs prior to the intervention period. After the 12-week intervention, participants of the intervention group were asked whether they were motivated to continue with the BeSt Age program and if the gamification element had influenced their motivation.

Assessments conducted among nursing home staff who delivered the physical exercise interventions included but were not limited to usability assessed using the System Usability Scale (calculated variable: score ranging from 0 to 100, with higher scores indicating better usability) [76], user experience using the AttrakDiff (calculated variables: pragmatic quality, hedonic quality divided into identity and stimulation, and attractiveness with scores ranging from –3 to 3) [77], affinity for technology, and app usage statistics. These considerations were based on implications derived from the RE-AIM (Reach, Effectiveness, Adoption, Implementation, and Maintenance) Framework [78]), which assesses reach (engagement among nursing home residents), effectiveness (health outcomes), adoption (nursing home staff willingness), implementation (consistency of use), and maintenance (long-term sustainability). Furthermore, nursing home staff were asked open-ended questions about reasons for offering physical activity programs and motivation to work with the BeSt Age app prior to the intervention period. After the 12-week intervention, they were additionally asked whether they enjoyed working with the app and whether they were motivated to use the BeSt Age app in the long term. Please refer to Table 1 for an overview of all primary and secondary outcomes. For all assessments, we used German language versions.

### Ethical Considerations

The study adhered to the guidelines and recommendations outlined in the CONSORT (Consolidated Standards of Reporting Trials) statement [79,80] as well as the CONSORT extension to cluster randomized trials [81]. The study was preregistered in the German National Register of Clinical Trials (DRKS00032349), and adhered to the guidelines outlined in the Helsinki Declaration. Ethical approval was granted from the ethics committee of the Karlsruhe Institute of Technology, Karlsruhe, Germany following a review of the study methodology and data protection measures. Written informed consent was obtained from all participants or, in the case of participants unable to consent, their legal guardians prior to the study. All participants were informed about the nature, procedures, and possible benefits and risks of the study, and could withdraw from the study at any time without consequence. Collected data were de-identified by assigning a unique ID to each participant, and the full set of data was accessible only to authorized members of the research team. Participants did not receive compensation.

## Results

### Characteristics of Participants (Nursing Home Residents) at Baseline

**Figure 5.**
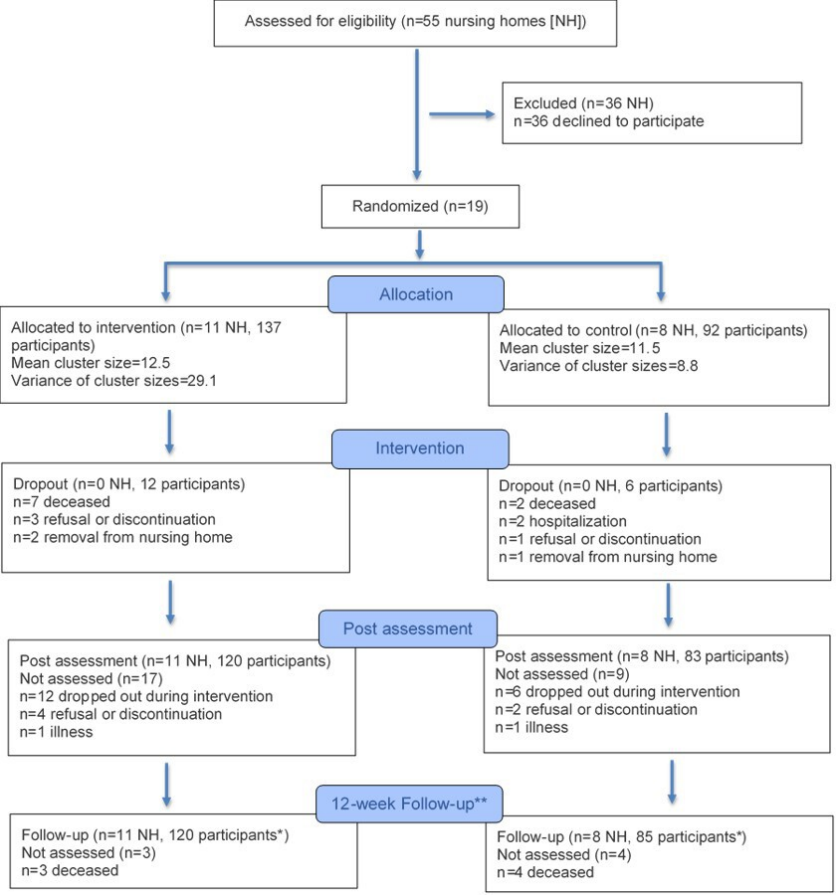
Flow diagram (modified according to Consolidated Standards of Reporting Trials). NH: nursing home. Single asterisk: Including some participants who did not attend postassessment (eg, due to illness and hospitalization). Double asterisk: Only number of falls assessed.

A total of 229 persons (171 females and 58 males; 137 intervention group and 92 control group) from 19 different nursing homes (11 intervention group and 8 control group) participated in the baseline assessment. Please refer to Figure 5 for a flow diagram detailing cluster allocation, resident recruitment, and attrition through T_2_. Mean age was 85.4 (SD 7.4) years, and participants rated their digital competency as low (mean score of 1.9, SD 1.1). The intervention group included a statistically significantly larger number of females, participants had a higher BMI, and more participants rated attending physical activity programs as important. There were no additional statistically significant differences between the intervention and control groups at baseline. Overall, 4 of 125 (3%) participants of the intervention group and 3 of 87 (3%) participants of the control group had a MoCA score of ≥26, indicating no cognitive impairment, while 40 of 125 (32%) participants of the intervention group and 33 of 87 (38%) participants of the control group had a score between 18 and 25, indicating mild cognitive impairment. Notably, 81 of 125 (65%) participants of the intervention group and 51 of 87 (59%) participants of the control group had a score ≤17, indicating moderate to severe cognitive impairment. Of note, MoCA data were missing for 17 participants, since we did not calculate a MoCA score for participants with visual or motor impairments or when German was not the mother tongue. With regard to mobility, 24 of 93 (26%) participants of the intervention group and 21 of 63 (33%) participants of the control group took 19 seconds or less to complete the Timed Up and Go test, indicating no mobility impairment, whereas 34 of 93 (37%) participants of the intervention group and 22 of 63 (35%) participants of the control group took between 20 and 29 seconds to complete, indicating impaired mobility, and 35 of 93 (38%) participants of the intervention group and 20 of 63 (32%) participants of the control group took ≥30 seconds, reflecting severe mobility impairment. Of note, 72 of 229 (31%) participants used no walking aid, 82 of 229 (36%) participants used a walker, and the remaining study participants used other tools, such as a walking stick or wheelchair. A total of 97 of 131 (74%) participants of the intervention group and 66 of 90 (73%) of the control group had no falls 6 months prior to the study baseline, 22 of 131 (17%) of the intervention group and 13 of 90 (14%) of the control group had 1 fall, and 12 of 131 (9%) participants of the intervention group and 11 of 90 (12%) of the control group had 2 or more falls. Please refer to Table 2 for an overview of participants’ characteristics, stratified by group allocation.

**Table 2.** Participant characteristics (nursing home residents) at baseline.

Variable	Intervention group (n=137)	Participants with missing information	Control group (n=92)	Participants with missing information	Total (N=229)	Participants with missing information	*P* value
Female sex, n (%)	111 (81)	—^a^	60 (65.2)	—	171 (74.7)	—	<.01
Age in years, mean (SD)	85 (7.6)	2	85.9 (7.2)	—	85.4 (7.4)	—	.31^b^
School education, n (%)							.14
No school leaving certificate	13 (10.1)	8	3 (3.3)	3	16 (7.3)	11	
Basic secondary school-leaving certificate	71 (55)	8	53 (59.6)	3	124 (56.9)	11	
Intermediate secondary school-leaving certificate	29 (22.5)	8	16 (18)	3	45 (20.6)	11	
Higher education entrance qualification (A-levels)	16 (12.4)	8	17 (19.1)	3	33 (15.1)	11	
BMI (kg/m^2^), mean (SD)	28.1 (5.8)	8	25.9 (5)	5	27.2 (5.6)	13	.02^b^
Digital competence score, mean (SD)	1.9 (1.1)	3	1.8 (1.2)	—	1.9 (1.1)	3	.43^b^
Quality of life and cognition							
EUROHIS^c^-Quality of life score, mean (SD)	30.5 (4)	8	29.9 (4.2)	1	30.3 (4.1)	9	.43^b^
Montreal Cognitive Assessment score, mean (SD)	13.9 (6.6)	12	15.1 (7.1)	5	14.4 (6.8)	17	.18^b^
Motor performance assessments							
Timed up and go test (time in seconds), mean (SD)	27.6 (11.7)	44	28.3 (16.5)	29	27.9 (13.8)	73	.53^b^
Grip strength (kg), mean (SD)	17.5 (6.7)	7	18.4 (8.7)	—	17.9 (7.6)^7^	7	.87^b^
10-meter walking test (m/s) at preferred pace, mean (SD)	0.61 (0.25)	25	0.58 (0.26)	10	0.60 (0.25)	35	.37^d^
Arm curl (number of curls in 30 seconds), mean (SD)	7.6 (4.5)	23	8.5 (5.8)	1	8 (5.1)	24	.23^b^
Berg Balance Scale score, mean (SD)	26.5 (16.3)	11	28.3 (16)	2	27.2 (16.2)	13	.41^b^
Short Falls Efficacy Scale score, mean (SD)	11.6 (4.5)	12	11.4 (4.2)	1	11.5 (4.4)	13	.75^b^
Number of falls (6 month before baseline), mean (SD)	0.54 (1.46)	6	0.60 (1.62)	2	0.57 (1.52)	8	.85^b^
Physical activity behavior and activities of daily living							
Life-Space Assessment in Institutionalized Settings score (participant-completed), mean (SD)	23.5 (14.7)	3	23.4 (13)	—	10.5 (5.2)	3	.77^b^
Life-Space Assessment in Institutionalized Settings score (nurse-completed), mean (SD)	28.1 (14.5)	6	29.2 (18.1)	13	28.6 (15.9)	19	.74^b^
Katz index, mean (SD)	3.7 (2)	13	4 (2)	11	3.8 (2)	24	.26^b^
Questions on physical activity							
Is it important for you to attend physical activity programs? n (%)							<.01
Yes	120 (88.9)	2	63 (70)	2	183 (81.3)	4	
No	15 (11.1)	2	27 (30)	2	42 (18.7)	4	
Are you motivated to attend the BeSt Age intervention? n (%)							
Yes	84 (62.7)	3	—	—	—	—	
No	18 (13.4)	3	—	—	—	—	
Undecided	32 (23.9)	3	—	—	—	—	
Did you participate in physical activity before the study? n (%)							.07
Yes	119 (90.2)	5	66 (81.5)	11	185 (86.9)	16	
No	13 (9.8)	5	15 (18.5)	11	28 (13.1)	16	

a Not applicable.

b Continuous, nonparametric data (Mann-Whitney *U* test).

c EUROHIS: European Health Interview Survey.

d Continuous, normally distributed data (2-tailed* t* test).

*P* values were calculated using the chi-square test (for categorical data) or the Mann-Whitney *U* test (for continuous, nonparametric data), or the 2-tailed *t* test (for continuous, normally distributed data). All percentages were computed relative to the sample with valid data (missing persons not included). An overview of the assessment tools and their scoring ranges is provided in Textbox 2.

Textbox 2. **Assessment tools, measures, and scoring criteria.**

**Digital competence score**

- Possible range 1‐5
- A lower score indicates lower competence

**EUROHIS (European Health Interview Survey)-Quality of life score**

- Possible range 8‐40
- A lower score indicates a worse quality of life

**Montreal Cognitive Assessment score**

- Possible range 0‐30
- A lower score indicates more severe cognitive impairment
- Persons with visual or motor impairments or for whom German was not the mother tongue were not considered

**Berg Balance Scale score**

- Possible range 0‐56
- A lower score indicates greater balance impairment

**Short Falls Efficacy Scale score**

- Possible range: 7‐28
- A higher score indicates more severe concern about falling

**Life-Space Assessment in Institutionalized Settings score**

- Possible range 0‐90
- A lower score indicates a greater degree of immobility

**Katz index**

- Possible range 0‐6
- A lower score indicates lower function and dependence

### Selected Results Derived From Nursing Home Staff at Baseline

We included 37 nursing home staff with a mean age of 51.7 (SD 11.5) years. Notably, 20 reported having a certification to deliver physical activity, and mean (SD) years of experience with delivering physical activity were 7.9 (SD 7.2). Qualitative analyses on assessments conducted among nursing home staff revealed that the app was rated as very good based on the System Usability Scale (mean score 87, SD 15) and was perceived as easy to administer, well designed, including a good selection of effective and varied exercises, and limiting the time for preparation of the physical exercise program delivery to a minimum. Suggestions for improvement were mentioned with regard to the duration of exercises or breaks between exercises and the level of intensity and difficulty of exercises, among others.

## Discussion

### Principal Findings

The novel, innovative BeSt Age app has been developed and implemented in a cRCT among 229 older adults residing in 19 nursing homes in Germany. The app offers a unique approach to delivering physical exercise in nursing homes. It contains a comprehensive exercise library and uses an in-built algorithm to select appropriate exercises. This selection process considers residents’ current motor and cognitive performance status, as well as their exercise preferences. Using the app, nursing home staff, regardless of their knowledge of physical exercise training, are enabled to deliver an individualized exercise program to nursing home residents. Of note, no or only minimal preparation is needed, which may be helpful to keep the burden for staff low.

The app contains a motivational feature, that is, credit points are awarded to a group for each completed training session. These points contribute to the growth and development of the group’s digital nursing home garden, thereby creating a tangible visual representation of collective physical activity engagement. This immediate feedback loop was designed to reinforce exercise behavior through visible achievement markers that participants could observe and understand. Indeed, a recent systematic review emphasized that mobile health gamification strategies, including but not limited to points, may improve motivation and participation in physical activities, albeit the review focused on gamification effectiveness in general populations [82]. Thus, it must be noted that the specific effectiveness of our garden-based point system for motivating exercise behavior in nursing home residents with or without cognitive impairment requires further empirical validation.

Results on the effectiveness of the BeSt Age app for nursing home residents, including but not limited to falls and fall-related outcomes, motor performance and activities of daily living, as well as quality of life, will become available in the near future. These outcomes were chosen based on prior research, which provided evidence of associations between physical activity and those variables or constructs in older adults. For example, a 3-month RCT using an individually tailored physical activity intervention in more than 300 nursing home residents with a mean age of 85 (SD 8) years revealed effects on physical activity level, balance assessed through the Berg Balance Scale, 10-meter walking speed, or functional leg muscle strength assessed through the timed chair stand test [31]. In addition, moderate-intensity physical activity is known to be related to lower fear of falling in women aged ≥60 years [83] and quality of life among older adults in care facilities [22]. Furthermore, an increased step count over a 12-week intervention in older adults with mild cognitive impairment was associated with improved MoCA test performance [84]. Finally, we also built on prior studies from our team showing effects of an app-delivered physical activity intervention on mobility or balance-related outcomes [44].

Upon demonstrating effectiveness, we plan to implement the app across nursing homes in Germany. It is conceivable that the BeSt Age app may be scaled up beyond a nursing home setting to be also used in community-based senior centers, rehabilitation programs, or independent living facilities. Collaboration with health insurance providers and policymakers could further facilitate app adoption, for example, as a reimbursable intervention for fall prevention and mobility improvement. We may also provide an English version of the app to facilitate future use in research and practice on an international scale. However, there may be challenges and limitations when exporting the app to non-German health care systems. To mitigate some of these, we designed the app in a way that allows utmost flexibility to nursing home staff; for example, nursing home staff can conduct sessions with one or more persons and adapt exercise sessions by skipping exercises during usage or adding and removing exercises before starting the program, as needed. Nevertheless, challenges may exist related to regulatory or legal frameworks, data protection and safety, country-specific staffing ratios and workflow integration that may make it more challenging (or alternatively even more simple) to use the app, or cultural compatibility, including but not limited to aspects related to nursing home staff or resident acceptance.

Overall, the BeSt Age app may be a viable solution for addressing the intention-behavior gap related to physical activity among older adults. While many nursing home residents recognize the benefits of exercise, most older adults fail to meet recommended physical activity levels, and various barriers prevent them from engaging in physical activity, including but not limited to lack of motivation or inadequate staff resources. Our app may help in bridging the intention-behavior gap by incorporating behavior change techniques (eg, goal setting, feedback mechanisms, and positive reinforcement through gamification), thereby possibly increasing adherence to physical activity routines. This may also have implications for clinical practice. Furthermore, many countries face nursing home staff shortages, and our app may be valuable in nursing homes, as it can support nursing home staff in delivering exercise training to residents without adding additional workload or requiring specific resources.

### Limitations

Our study has some limitations. Although an a priori power analysis was conducted, the sample size justification is built on assumptions from ANOVA that may not hold due to the cluster design and unvalidated ICC estimates, which may raise concerns about type II errors. Furthermore, the intervention group performed 2 sessions per week, and the duration for each session was only 25‐30 minutes, which is rather low relative to World Health Organization and other guidelines for older adults. We decided on this duration based on experience from prior research and feedback we received from nursing home staff, indicating that any session duration longer than 30 minutes might not be realistic. Nevertheless, it must be acknowledged that both the duration and frequency of the intervention might have been too low to yield any effects on our primary and secondary outcome measures.

Another limitation pertains to the fact that we have not collected any cost-effectiveness or implementation-economics metrics to inform potential future scale-up decisions. Furthermore, we used several subjective outcomes that may be prone to assessment bias. However, to mitigate bias, we only used tests and questionnaires that have been validated and used in other studies in Germany or with the same or comparable target populations before. Also, in our study, blinding of participants (ie, nursing home residents and staff) was not possible, which may increase bias and compromise the validity of results. In addition, randomization at the facility level, combined with residents providing consent only after allocation, may introduce a potential selection bias. However, to address initial nonparticipation, multiple recruitment attempts within each facility over an extended period were carried out that allowed residents time to reconsider and potentially reduced selection bias.

Moreover, our analysis plan follows intention-to-treat principles, where facilities remain in their allocated groups regardless of individual resident participation rates, thus maintaining the integrity of the randomization. Furthermore, during interface design, we did not follow specific accessibility standards for residents with visual or motor limitations, since the app was designed to be used by nursing home staff. However, such adaptations may need to be made in the future. In addition, we did not implement a systematic adverse event capture system in our study. However, through our regular communication with facility coordinators across all participating nursing homes, no adverse events related to the intervention were reported to our research team.

Finally, we did not prespecify minimal clinically important difference thresholds for our primary outcomes, including the EUROHIS-QOL and Berg Balance Scale, and our sample size calculations were based on detecting statistically significant differences rather than clinically meaningful changes. This decision was made due to limited established minimal clinically important difference benchmarks for these instruments, specifically among nursing home residents with cognitive impairment. In addition, the heterogeneity of our target population in terms of baseline functional capacity and cognitive status made it challenging to establish universally applicable clinical significance thresholds.

### Conclusions

We postulate that the BeSt Age app may be used to promote physical activity and prevent falls in nursing home residents, thereby ultimately contributing to maintaining quality of life and overall well-being in this vulnerable population. Many older adults face challenges in using digital interventions. By designing the app to be intuitive and staff-assisted, it can overcome digital literacy barriers, ensuring accessibility for residents with or without cognitive impairments. Future directions may include investigation of the long-term adherence to our digital exercise program, exploration of the role of gamification in sustained motivation, examination of the potential of the app to save time or decrease burden for nursing home staff, and conduct of comparative studies with other digital interventions to assess relative effectiveness.

## Supplementary material

10.2196/74174 Multimedia Appendix 1 Examples for exercises.

## ABBREVIATIONS

CG: control group
COM-B: Capability, Opportunity, Motivation, and Behavior
CONSORT: Consolidated Standards of Reporting Trials
cRCT: cluster randomized controlled trial
EUROHIS: E
EUROHIS-QOL: European Health Interview Survey-Quality of Life
ICC: intraclass correlation coefficient
IG: intervention group
InCoPE: Individualized Cognitive and Physical Exercise
LSA-IS: Life-Space Assessment in Institutionalized Settings
MoCA: Montreal Cognitive Assessment
RE-AIM: Reach, Effectiveness, Adoption, Implementation Maintenance
