# Supplementary material for: Promoting Physical Activity and Preventing Falls Among Older Adults in a Nursing Home Setting: Protocol for Development and Implementation of the BeSt Age Mobile App
Source: JMIR Res Protoc. 2025 Oct 6;14:e74174. doi: 10.2196/74174 (PMC12500227; doi:10.2196/74174)

Example for warm-up exercise

Walking in place while being seated

<u>Starting position:</u>

Sit upright on a chair without leaning against the backrest, maintaining a slight distance from it. Both feet should be flat on the floor, with knees bent at a 90-degree angle.

<u>Exercise description:</u>

Lift your right leg until your foot is off the floor. Lower it in a controlled manner, placing the heel down first, followed by the rest of the foot. Repeat the movement with your left leg. Repeat the sequence until the exercise is complete.

<u>Screenshot of the app:</u>

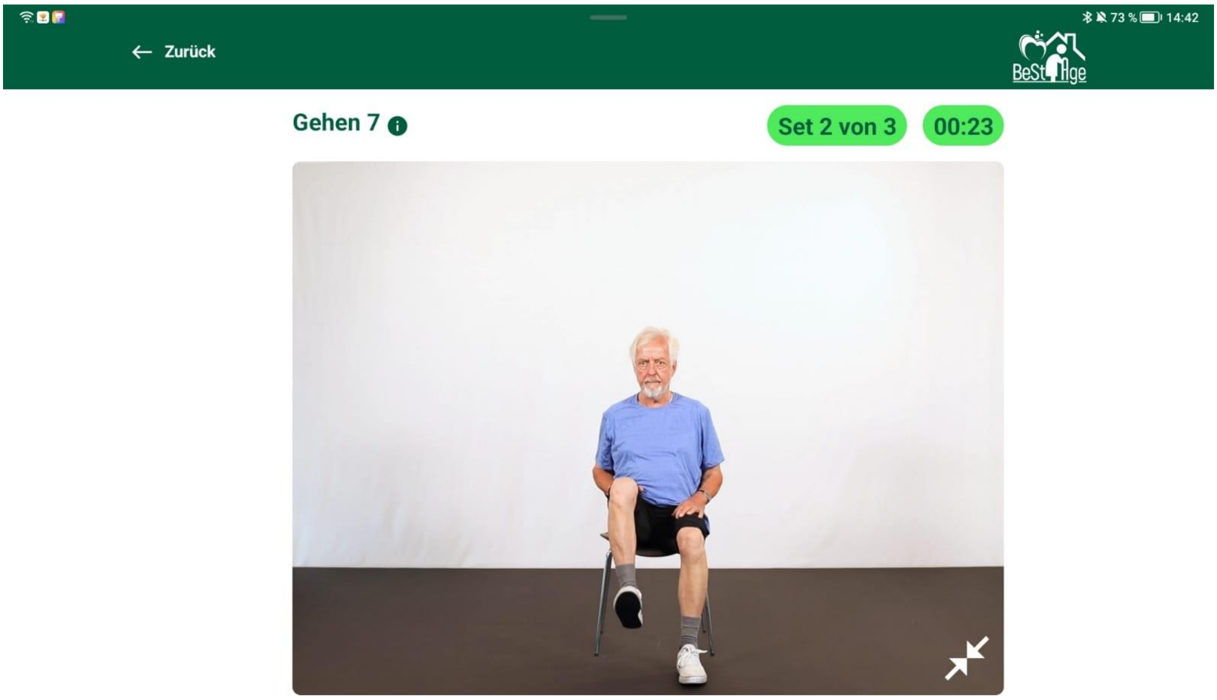

Example for main exercise

Weight shift

Starting position:

Stand upright behind a chair. Your legs should be parallel and about hip-width apart. Both hands rest on the backrest of the chair

Exercise description:

Slowly shift your weight slowly onto your right leg. If possible, lift your left leg slightly to the side. Hold the position briefly, then return to the center in a controlled manner. Throughout the movement, keep your hands on the backrest of the chair for support. Repeat the sequence with the left leg. If there is any instability during the movement, the exercise can be performed while seated.

Screenshot of the app:

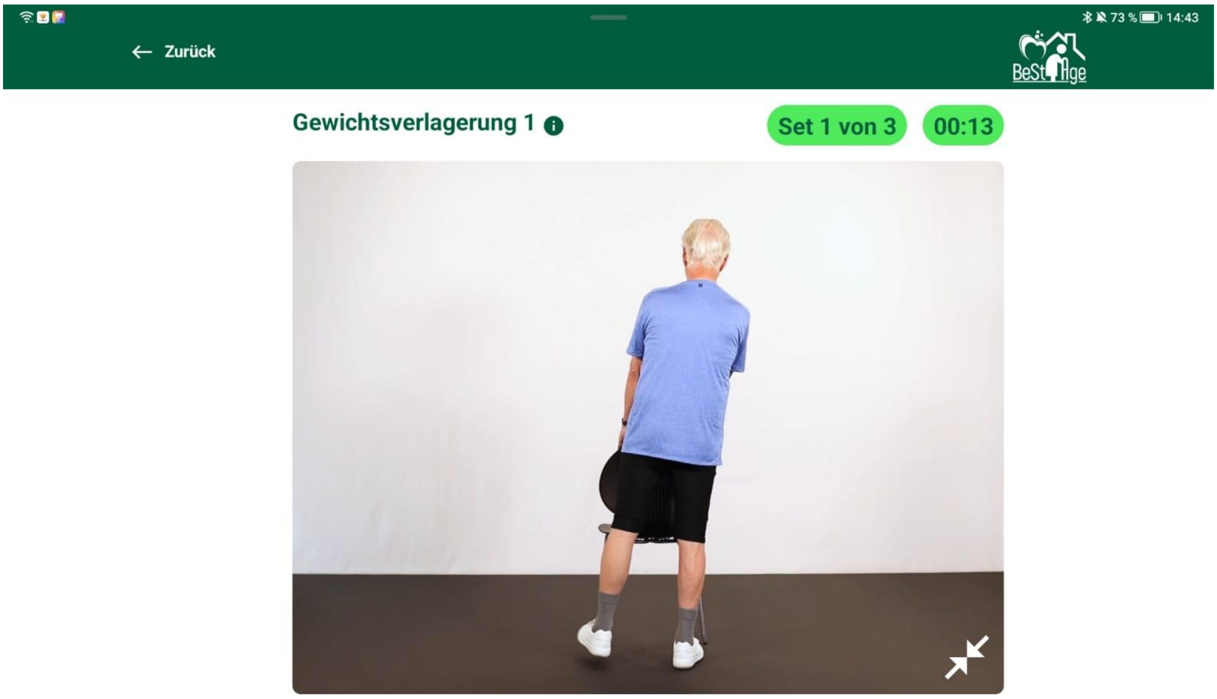

Example for main exercise:

Cognition and movement

<u>Starting position:</u>

Sit upright on a chair without leaning against the backrest, maintaining a slight distance from it. Position your legs hip-width apart, with your feet flat on the floor and knees bent at a 90-degree angle.

<u>Exercise description:</u>

Lift your right leg and guide the knee outward so that the right foot moves toward the left knee. Lower your left hand to touch the right foot. Return to the starting position. Then switch sides, bringing together the right hand and left leg. Cognitive stimulation: While performing the exercise, name different types of fruits and vegetables.

<u>Screenshot of the app:</u>

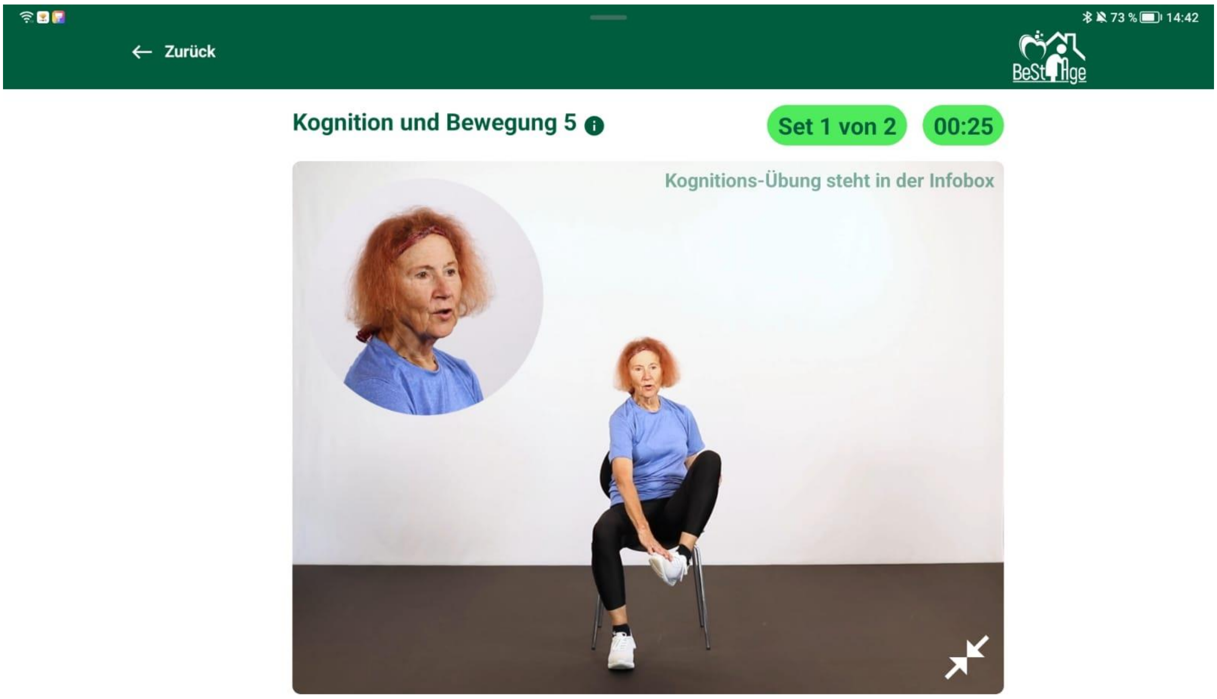

Example for cool-down exercise

Facial gymnastics

Starting position:

Sit upright on a chair without leaning against the backrest, maintaining a slight distance from it.

Exercise description:

1) Frown your forehead three times, then relax

2) Lift your nose upward three times, then relax

3) Raise your eyebrows three times, then relax

4) Form your lips into a kiss shape three times, then relax

5) Open your mouth wide three times, then relax

6) Facial massage: Gently massage your face using your fingertips. Begin at the temples and move along the cheekbones toward the jawline

Screenshot of the app:

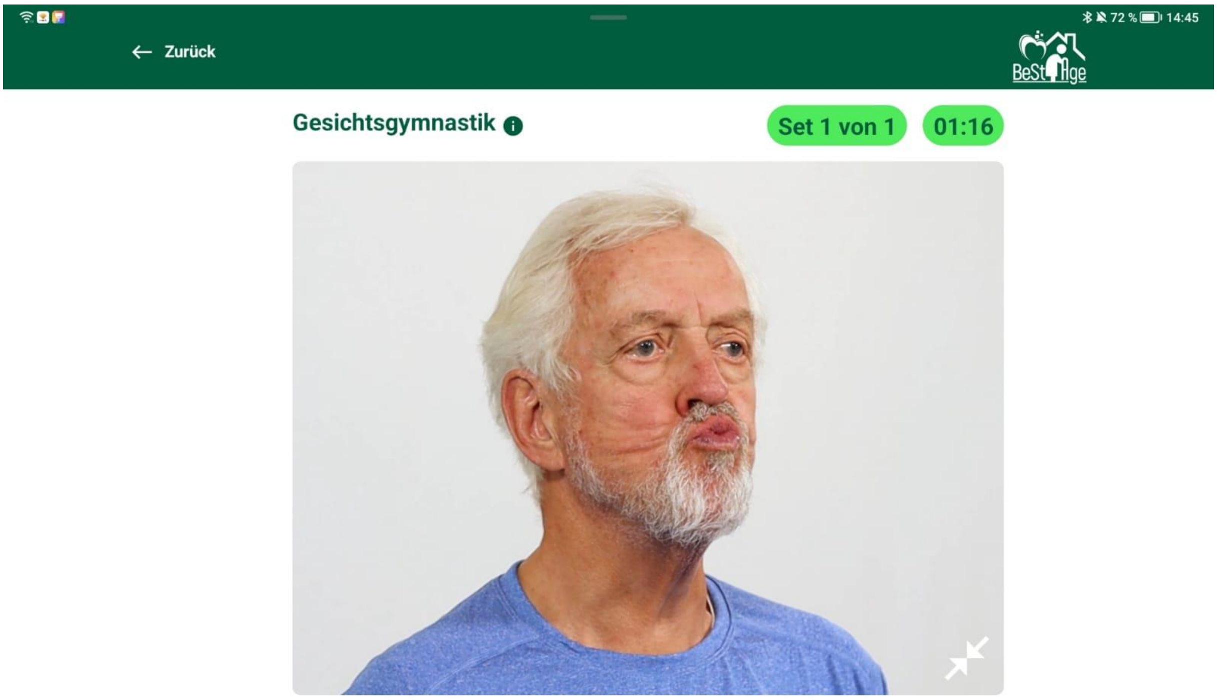

Supplement: Multimedia Appendix 1 [file resprot-v14-e74174-s001.pdf]
